# FoxO Transcription Factor Regulate Hormone Mediated Signaling on Nymphal Diapause

**DOI:** 10.3389/fphys.2018.01654

**Published:** 2018-11-20

**Authors:** Zhen-Juan Yin, Xiao-Lin Dong, Kui Kang, Hao Chen, Xiao-Yan Dai, Guang-An Wu, Li Zheng, Yi Yu, Yi-Fan Zhai

**Affiliations:** ^1^Institute of Plant Protection, Shandong Academy of Agricultural Sciences, Jinan, China; ^2^College of Agriculture, Yangtze University, Jingzhou, China; ^3^School of Life Sciences, Sun Yat-sen University, Guangzhou, China; ^4^College of Life Sciences, Shandong Normal University, Jinan, China

**Keywords:** FoxO, RNAi, nymphal diapause, *Laodelphax striatellus*, RNA-sequencing (RNA-Seq)

## Abstract

Diapause is a complex physiological adaptation phenotype, and the transcription factor Forkhead-box O (FoxO) is a prime candidate for activating many of its diverse regulatory signaling pathways. Hormone signaling regulates nymphal diapause in *Laodelphax striatellus*. Here, the function of the *FoxO* gene isolated from *L. striatellus* was investigated. After knocking-down *LsFoxO* in diapausal nymphs using RNA interference, the titers of juvenile hormone III and some cold-tolerance substances decreased significantly, and the duration of the nymphal developmental period was severely shorted to 25.5 days at 20°C under short day-length (10 L:14 D). To determine how *LsFoxO* affects nymphal diapause, analyses of RNA-sequencing transcriptome data after treatment with *LsFoxO*–RNA interference was performed. The differentially expressed genes affected carbohydrate, amino acid and fatty acid metabolism, and phosphatidylinositol 3-kinase/protein kinase B signaling pathway. Thus, *LsFoxO* acts on *L. striatellus* nymphal diapause and is, therefore, a potential target gene for pest control. This study may lead to new information on the regulation of nymphal diapause in this important pest.

## Introduction

Insects have evolved multiple strategies to adapt to environmental changes, such as diapause and migration. Diapause enables insects to decrease metabolism, arrest development and increase stress resistance under unfavorable conditions, and it is regulated by external environmental signals and internal genetic factors (Denlinger, 2002, 2008). Most insects rely on photoperiod and temperature signals to reach diapause, and there are several regulatory features of diapause, such as hormonal molecular regulation, the circadian clock and energy utilization (Eizaguirre et al., 1998; Anspaugh and Roe, 2005; Xu et al., 2012; Kauranen et al., 2016; Li et al., 2017). In addition, some cold tolerance substances accumulate and key enzymes catalyze, such as trehalase (TRE), sorbitol dehydrogenase (SDH), and pyruvate kinase (PK), etc., to improve the cold tolerance and overcome severe winter environments, (Rozsypal et al., 2013; Zhai et al., 2016). In addition, diapause typically occurs at a specific developmental stage for each species, such as the embryo, larvae/nymph, pupae or adult (Kostal, 2006; Williams et al., 2006; Zhang et al., 2013; Fan et al., 2017). Larval/nymphal diapause occurs because many insect species overwinter as larvae/nymph, in which the molting processes is usually arrested, and some related signaling pathways are suppressed, such as hormone and energy metabolism (Hahn and Denlinger, 2011; Jindra et al., 2013).

The Forkhead box (Fox) proteins form a family of transcription factors that has several subclasses. The member proteins are diverse but are characterized by a conserved DNA-binding domain. FoxO is the main transcriptional effector of the insulin signaling pathway and is normally suppressed in the presence of insulin (Martins et al., 2016). The insulin signaling pathway plays a critical role in regulating diapause in some invertebrates, such as *Caenorhabditis elegans*, *Drosophila melanogaster*, and *Culex pipiens* (Junger et al., 2003; Sim and Denlinger, 2008; Matsunaga et al., 2018). High insulin levels activate the phosphatidylinositol 3-kinase/protein kinase B (PI3K/Akt) pathway, which in turn phosphorylates FoxO, promoting FoxO inactivation. In *C. pipiens*, the shutdown of insulin signaling prompts the activation of downstream FoxO and leads to the adult diapause phenotype (Sim et al., 2015).

The small brown planthopper, *Laodelphax striatellus* Fallén (Hemiptera: Delphacidae), is a notorious pest in a variety of graminaceous crop systems, including rice, wheat, corn and barley (Kisimoto, 1989). It causes serious damage to plants due to the transmission of viruses associated with plant diseases, such as rice stripe virus and rice black-streaked dwarf virus (Otuka, 2013). *L. striatellus* exhibits more cold resistance than other rice planthopper species, *Nilaparvata lugens* (Stål) and *Sogatella furcifera* (Horváth), and the most *L. striatellus* northern populations showed the highest diapause incidence and a longer critical photoperiod (Hou et al., 2016). *L. striatellus* nymphal diapause has been studied under different laboratory conditions; however, little research has focused on the molecular regulatory mechanisms related to nymphal diapause (Kisimoto, 1989; Wang et al., 2014). In our previous study, we determined the 4th-instar nymph as the main diapause stage through investigation under field and laboratory conditions (Zhai et al., 2018). Here, we characterized the functions of the *LsFoxO* gene in nymphal diapause. The injection of *dsLsFoxO* significantly altered the levels of some cold-tolerance substances, metabolic enzymes activities and hormone titers, and the duration of diapause in nymphs was shortened.

## Experimental Section

### Ethics Statement

The small brown planthopper, *Laodelphax striatellus* is an economically important pest insect in East Asia, which attacks a wide range of graminaceous crops. The field studies did not involve endangered or protected species, and no specific permissions were required for our research activities in these locations.

### Insect Rearing

We obtained the original *L. striatellus* colony from the Shandong Rice Research Institute (SRRI; Shandong, China) in 2010. These insects were reared on fresh rice seedlings and maintained in the laboratory at 25 ± 1°C under a 16 L: 8 D photo regime and 70–80% relative humidity. Newly hatched 1st instar nymphs were reared on fresh rice seedlings at 20°C under long day-length (16 L: 8 D), which resulted in all 4th instar nymphs individuals continuing through direct development (non-diapause). On the contrary, newly hatched 1st instar nymphs were reared at 20°C under short day-length (10 L:14 D), resulting in substantially all 4th instar nymphs individuals entering nymphal diapause, and developmental delay often characterized nymph population diapause (Wang et al., 2014; Zhai et al., 2018).

### The Cloning and Sequence Analyses of *LsFoxO*

The Total RNA Kit II (Omega Bio-Tek, Norcross, GA, United States) was used to isolate the total RNA from larvae and pupae of *L. striatellus*, and first strand cDNA synthesis was performed using the 1st Strand cDNA Synthesis Kit (TaKaRa, Tokyo, Japan). Two pairs of intermediate fragments primers were designed from the *L. striatellus* transcriptome database by local blast (Table 1). The full-length complementary DNA (cDNA) of *LsFoxO* was cloned using the rapid amplification of cDNA ends RACE kit (Takara, Japan), and the evolutionary analyses were conducted using MEGA software, version 4.0.

**Table 1:** Primers used in this study.

Primer name	Primer sequences (5′ - 3′)
**Intermediate fragments primers**
*LsFoxO*-F1	GATAGGTGAGATGTCCGAGTGA
*LsFoxO*-F2	GCAGTAGTTGGTGTTGGTTGT
*LsFoxO*-R1	GCTGCTGCGTGAAGTTGAA
*LsFoxO*-R2	TGATGAGGTCGGCGTAGGA
**For cDNA cloning**	
5′- *LsFoxO*-1	TGTGTGATGAGGTCGGCGTAGGAGA
5′- *LsFoxO*-2	CAAATCCTCACTCGGACATCTCAC
3′- *LsFoxO*-1	GCCTCTGCCTGGCTTTCAACTAA
3′- *LsFoxO*-2	TCCGCCAGCGACAACGGTGAT
**For RT-PCR and real-time PCR**
Q *LsFoxO*-F	TCTGCCTGGCTTTCAACTAA
Q *LsFoxO*-R	CCGAGTCGCATCGTCTGT
*EF-1*-F	CCTTACCCATGTTGGATGCTTATT
*EF-1*-R	TGCTTCTGTCTTCCTCTTTCTTCC
*ARF*-F	TTGGACAGTATCAAGACCCATC
*ARF*-R	GCAGCAATGTCATCAATAAGC
**For dsRNA synthesis**	
*dsLsFoxO*-F	ggatcctaatacgactcactataggGGAACGGCCTGGATGCTA
*dsLsFoxO*-R	ggatcctaatacgactcactataggCTGCGGGTATGAAGGTGAG
*dsGFP*-F	ggatcctaatacgactcactataggACGTAAACGGCCACAAGTTC
*dsGFP*-R	ggatcctaatacgactcactataggTGTTCTGCTGGTAGTGGTCG


### Quantitative Real-Time PCR Analysis

The primers used for real-time PCR are listed in Table 1. The synthesized first-strand cDNA was amplified by PCR in 10 μL reaction mixtures using a Light Cycler 480 system (Roche, United States), and *ADP-ribosylation factor* (*ARF*) and *elongation factor-1* (*EF-1*) genes were used as an internal standard (Wan et al., 2014). The quantitative variation was calculated using three independent biological samples by a relative quantitative method (2^-ΔΔCT^).

### Western Blotting

The proteins were separated using 12% SDS-PAGE gel and transferred to PVDF membranes (0.4 μm; EMD Millipore, Hayward, CA, United States), and the membranes were immunoblotted with anti-*LsFoxO* serum (1:3000) prepared by our laboratory. IgG goat anti-mouse and goat anti-rabbit antibodies conjugated with HRP were used for secondary antibodies (1:5000, Abcam, Cambridge, United Kingdom), and the membranes were visualized by ECL.

### RNA Interference and Sampling

The dsRNA of *LsFoxO* was produced using the T7 RiboMAXTM Express RNAi System (Promega, Sunnyvale, CA, United States). After synthesis, *dsLsFoxO* (MF197906, 431 bp) and *dsGFP* (DQ389577, 495 bp) were quantified by an ultramicro-spectrophotometers (NanoDrop 2000, Thermo Fisher, Scotts Valley, CA, United States) and were maintained at -80°C until use (Table 1). The sequence was verified by sequencing (Sangon Biotech, Shanghai, China). Before injection, the dsRNA and phenol red solution were mixed for observations. Under carbon dioxide anesthesia, nymphs were immobilized on the agarose injection plate with the ventral side upward, under CO_2_ anesthesia. The purified *dsLsFoxO* and *dsGFP* were slowly injected on one side of the metathorax using the Nanoject II (Drummond, Broomall, PA, United States). The injected individuals were placed in a glass tube (length: 200 mm; diameter: 25 mm) on fresh rice seedlings for further observation. Data on developmental duration were recorded every day until the adult emerged.

### Assessment of Metabolic Enzyme Activities and Biochemical Substances

To clarify the physiological adaptation of first day fourth-instar diapause nymphs with *dsLsFoxO* or *dsGFP* treated. Glycogen (MAK016) and triglyceride (TR0100) were measured by commercial kits (Sigma-Aldrich Co., LLC., United States), and trehalose (K-TREH) was measured by commercial kits (Megazyme, Ireland). Some cold tolerance-related metabolic enzymes, such as TRE, SDH and PK were quantified. The activities of metabolic enzymes were measured by commercial kits (Suzhou Comin Biotechnology Co., Ltd., Suzhou, China), and the absorbance of TRE, SDH and PK were measured at 340 nm.

### Quantitative Determination of Hormone

*Laodelphax striatellus* samples were separately ground in grinder and ultra sonicated with methanol and isooctane. After centrifugation at 12,000 *g* for 10 min, the upper layer was transferred into a test tube, the ultrasound-assisted extraction was repeated twice. The combined extracts were evaporated to dryness in a 40°C water-bath under a stream of nitrogen. The residue was reconstituted in methanol, then transferred to injection vials and analyzed using HPLC-MS/MS, (Agilent 6420; Waldbronn, Germany). JH III was separated using gradient elution and the hormone titer was expressed as ng per mg body weight.

### Transcriptomic Analyses

The total RNA was extracted using the E.Z.N.A.^®^ Total RNA Kit II (Omega Bio-Tek, Norcross, GA, United States) according to the manufacturer’s instructions. To obtain ideal gene expression information after the RNAi, first day fourth-instar diapause nymphs were injected with *dsLsFoxO* or *dsGFP*, and after 48 h, the samples were used for transcriptomic analyses. The genes differentially expressed between the two samples were identified using an algorithm as previously described (Schulze et al., 2012). Each cDNA library was sequenced using the Illumina sequencing platform (Hiseq 2500; Illumina, Hayward, CA, United States) according to the manufacturer’s instructions.

### Statistical Analyses

The statistical analyses were performed using SPSS 17.0 software, differences between treatments levels were examined using ANOVA, followed by Tukey’s analysis.

## Results

### Isolation and Characterization of *LsFoxO* cDNA

The full-length *LsFoxO* sequence was 2,766 bp (GenBank accession No: MF197906) and had an open reading frame (ORF) of 1,275 bp, which encoded a protein of 424 amino acids with a predicted mass of approximately 46.41 kDa and an isoelectric point of 7.04, with a 5′-untranslated region (UTR) of 781 bp and a 3′-UTR of 710 bp (Supplementary Figure 1).

**FIGURE 1:**
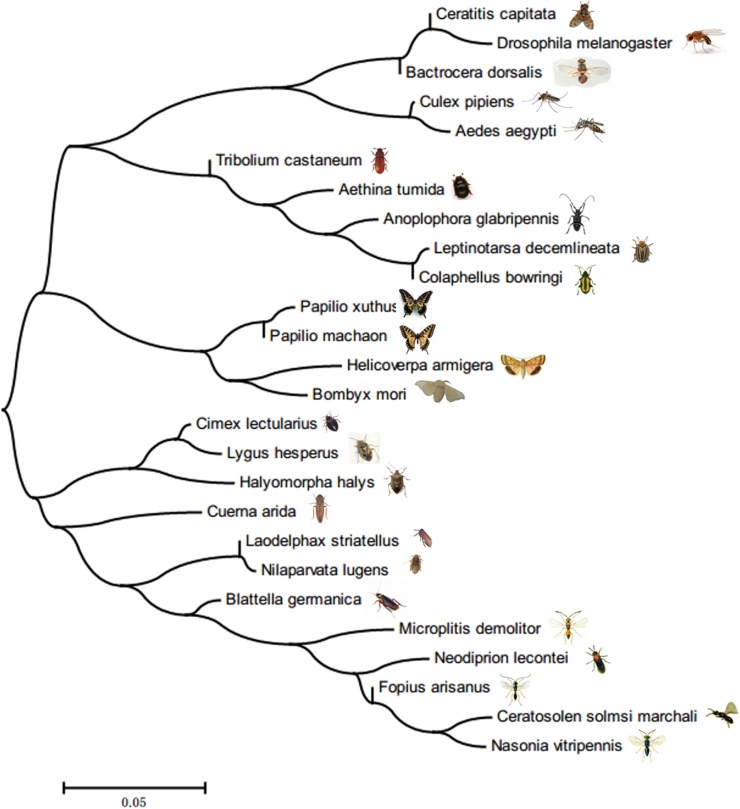
Neighbor-Joining phylogenetic tree based on *LsFoxO* amino acid sequences.

A phylogenetic tree was constructed based on the full-length sequences of known FoxO genes from insects and other organisms (Figure 1). The BLAST results showed that the amino acid sequence of *LsFoxO* had the greatest similarity to FoxO from *Nilaparvata lugens* (Hemiptera) (88%) (XP_022196038), *Halyomorpha halys* (Hemiptera) (69%) (XP_014290452), *Cimex lectularius* (Hemiptera) (69%) (XP_014254467), *Helicoverpa armigera* (Lepidoptera) (58%) (XP_021186671), *Pieris rapae* (Lepidoptera) (56%) (XP_022130764).

### The Spatiotemporal Expression of *LsFoxO*

**FIGURE 2:**
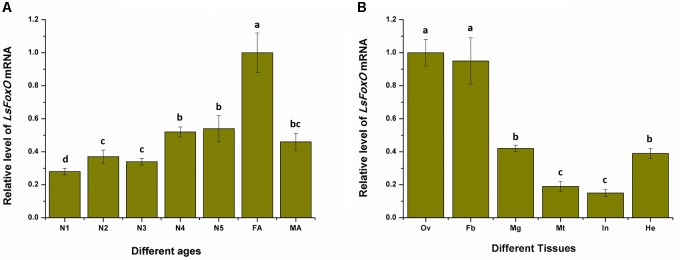
Expression of *LsFoxO* gene at different developmental stages **(A)** and various tissues **(B)** were determined by qRT-PCR. **(A)** N1–N5, nymph of 1st to 5th; FA, second day brachypterous female adult; MA, second day brachypterous male adult. **(B)** Ov, ovary; Fb, fat body; Mg, midgut; Mt, malpighian tube; In, integument; He, hemolymph. Data represent mean values ± SEM (*n* = 3), and the different letters means significant difference (*p* < 0.05, Tukey’s *post hoc* test).

To determine whether *LsFoxO* was present during developmental stages and in various tissues in the female adult *L. striatellus*, total RNA from each sample was isolated. We used qRT-PCR to characterize the *LsFoxO* gene’s expression pattern in all of the developmental stages. The *LsFoxO* mRNA expression level was high in the 4th–5th instar nymphal period, but the expression level was higher in the female adult period (Figure 2A). The expression of *LsFoxO* mRNA was investigated in various tissues in adult females. *LsFoxO* was highly expressed in the fat body, hemolymph and ovary, with lower expression levels in the integument, Malpighian tube and midgut (Figure 2B).

### Effect of Knocking Down *LsFoxO* on Nymphal Performance

**FIGURE 3:**
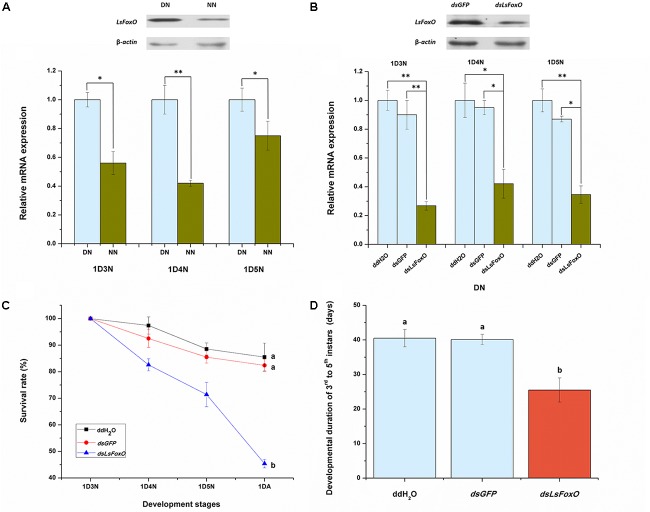
Relative mRNA expression of *LsFoxO* gene were determined by qRT-PCR. **(A)** *LsFoxO* transcript levels in diapause and non-diapause nymphs, at 1st day third instar nymph (1D3N), 1st day fourth instar nymph (1D4N) and 1st day fifth instar nymph (1D5N). Inset shows Western blotting analysis of LsFoxO protein levels at 1D4N. **(B)** The transcript levels of *LsFoxO* after RNAi in diapause nymphs. Inset shows the LsFoxO protein levels injected with *dsRNA* at 72 h postinjection after 1D4N. **(C)** The survival rates of diapause nymphs from 3rd instar to the initiation of adult stage. **(D)** The developmental duration of diapause nymphs. Three replicates were conducted, with the data presented as mean ± SEM. Significant differences between treatment and control are indicated with asterisks (^∗^*p* < 0.05; ^∗∗^*p* < 0.01), and the different letters means significant difference (*p* < 0.05, Tukey’s *post hoc* test).

To evaluate the effects of an *LsFoxO* knockdown on nymphal diapause, we used a microinjection-based RNA interference (RNAi) method. Before analyzing the RNAi efficiency, we detected the *LsFoxO* mRNA expression level in diapausal nymphs (DNs) and non-diapausal nymphs (NNs), including 1st day 3rd instar nymph (1D3N), 1st day 4th instar nymph (1D4N) and 1st day 5th instar nymph (1D5N). The *LsFoxO* mRNA expression levels were significantly up-regulated in DNs, and the protein levels were also increased in DNs at 1D4N (Figure 3A). At 48 h after an injection of *dsLsFoxO*, the expression of *LsFoxO* decreased by 73.19, 57.90, and 65.40% in the three different periods, respectively, compared with an injection of ddH_2_O. Western blot analyses showed that the LsFoxO protein levels also decreased after injections of *dsLsFoxO* (Figure 3B). These results indicated that the RNAi was effective.

The injection of *dsLsFoxO* significantly decreased the nymphal survival rate to 45.4%, and the nymphal period from 3rd to 5th instar was significantly shortened after injection. By contrast, over 82% of the nymphs injected with water or *dsGFP* survived (Figure 3C). The average duration to adult eclosion of nymphs injected with *dsLsFoxO* was 25.5 days, which was significantly shorter than the mean periods of other treatments at 20°C under short day-length conditions (10 L:14 D) (Figure 3D).

### Assessment of Physiological and Biochemical Changes

**FIGURE 4:**
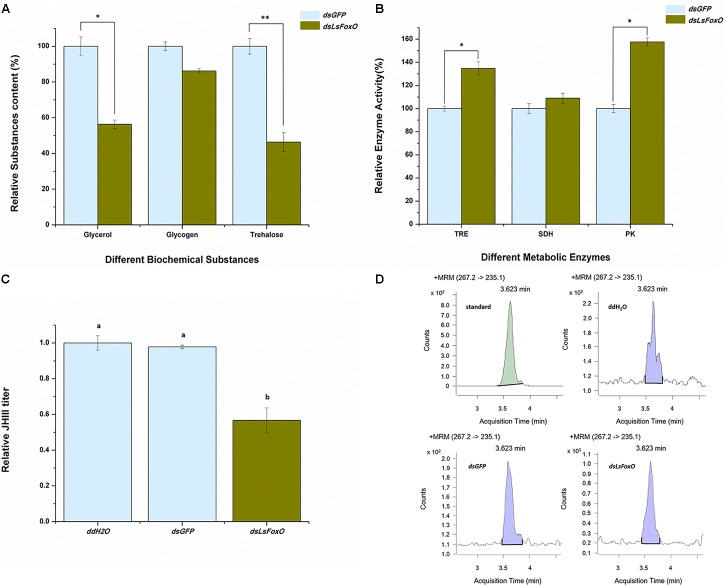
Relative physiological and biochemical changes. **(A)** Relative substance content, **(B)** Relative enzyme activity, and **(C)** the JH III titer were analyzed at 72 h post-injection by different treatment at 1D4N. **(D)** The representative chromatograms of JH III. Three replicates were conducted, with the data presented as mean ± SEM. Significant differences between treatment and control are indicated with asterisks (^∗^*p* < 0.05; ^∗∗^*p* < 0.01), and the different letters means significant difference (*p* < 0.05, Tukey’s *post hoc* test).

Diapause regulates several physiological and biochemical mechanisms, particularly modifying the activities of some cold-tolerance substances and metabolic enzymes. At 72 h after an injection of *dsLsFoxO*, the glycerol and trehalose levels significantly decreased (43.63 and 53.62%, respectively) (Figure 4A), the enzymatic activities of TRE and PK significantly increased (34.79 and 57.62%, respectively) (Figure 4B), and the juvenile hormone (JH) III titer indicated that the hormone levels were significantly decreased (Figures 4C,D).

### Global Changes at the Transcript Level After *LsFoxO* RNAi

**FIGURE 5:**
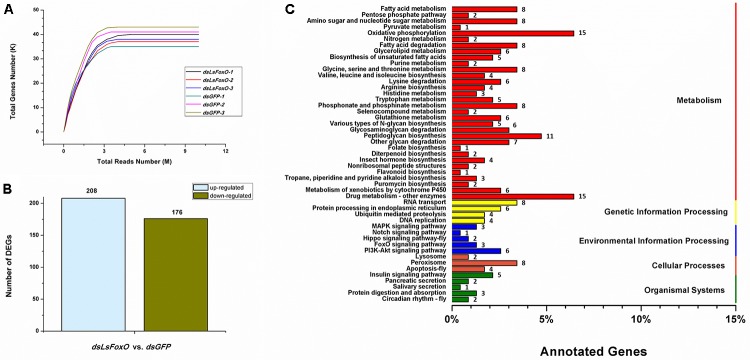
Transcriptomic analysis of the DEGs data in *LsFoxO* knockdown diapause nymphs. **(A)** Simulated diagram of saturation test of sequencing data. X axis indicates the number of reads (10^6^), Y axis indicates the number of detected genes (10^3^) and FPKM ≥ 0.1. **(B)** Number of significantly changed annotated DEGs, the conditions for genes was FDR ≤ 0.01 and FC ≥ 2. **(C)** The distribution of pathways of DEGs annotated in the KEGG data library.

A total of 38,547,651 clean pair-end reads were generated by Illumina sequencing and were *de novo* assembled into 42,273 unigenes, with an N_50_ length of 1,357 bp (Supplementary Table 1). The saturated gene number achieved with the increase in sequenced reads indicates that sufficient and effective information was applied in this study (Figure 5A). *L. striatellus* lacks a reference genome; therefore, 31,254 unigenes were annotated from the Clusters of Orthologous Groups (COG, 10,671), GO (10,083), Kyoto Encyclopedia of Genes and Genomes (KEGG, 9,794), EuKaryotic Orthologous Groups (KOG, 18,483), Protein family (Pfam, 15,526), SwissProt (9,384) and NR (27,325) databases using a cut-off *e*-value of 10^-5^ (Supplementary Table 2). The differentially expressed genes (DEGs) following RNAi treatment were analyzed according to the gene expression level (FPKM). Based on the DEG analysis, 384 genes had significantly different expression levels between the *dsLsFoxO-* and *dsGFP*-treated libraries, including 208 up- and 176 down-regulated genes (Figure 5B). According to the KEGG analysis, most of the DEGs correlated with metabolic and environmental information processes, including carbohydrate metabolism, amino acid metabolism, fatty acid metabolism and the PI3K-Akt signaling pathway (Figure 5C).

## Discussion

Diapause usually occurs during a specific stage, such as embryo, larvae/nymph, pupae or adult stages (Jiang et al., 2010; Liu et al., 2010; Kobayashi et al., 2014). Some research have found that diapause occurs in several nymphal stages of *L. striatellus*, and which varies with geographic location (Wang et al., 2014; Hou et al., 2016). In our previous study, we discovered that the overwintering diapause occurred in 3rd to 5th instars, with the 4th instar nymph being the predominant diapause stage (Zhai et al., 2018). Diapause is a complex physiological response process with many regulatory features, number of genes, proteins, and metabolites that are differentially expressed in diapause (Zhang et al., 2013). Diapause is regulated by the JH, and ecdysone in diverse species. A high JH titer inhibits ecdysone secretion during diapause maintenance, and the JH titer decreases significantly during late diapause. Hormone signaling regulates nymphal diapause in *L. striatellus* (Zhai et al., 2017). In *D. melanogaster*, JH and ecdysone synthesis are regulated by the insulin signaling pathway (Colombani et al., 2005; Tu et al., 2005), and insulin signaling is a regulator of diapause (Sim and Denlinger, 2013). FoxO is a well-known regulator of life span extension (Martins et al., 2016), insulin activates p-Akt levels repress FoxO activity, which activate other cross-talk genes that promote the insects’ development. In contrast, a low level of insulin signaling represses PI3K/Akt and increases the FoxO activity, which regulates life-span extension and generates the diapause phenotype (Sim et al., 2015). High levels of p-Akt fail to phosphorylate FoxO through PRMT1-mediated methylation, blocking FoxO phosphorylation reduces FoxO protein degradation, thus promoting the accumulation of FoxO in brains, which leads to pupal diapause in *Helicoverpa armigera* (Zhang et al., 2017). The overexpression of FoxO during early larval stages inhibits development and extends life-spans. Our results, in which *LsFoxO* mRNA expression and protein levels were significantly up-regulated in diapause nymphs, corroborates previous findings (Figure 3A).

After evaluating the spatiotemporal expression of the *LsFoxO* gene in *L. striatellus* using qRT-PCR, we found that higher expression levels were detected in 4th–5th instar nymphs and the female adults. FoxO is expressed during all of the developmental stages in other insects (Hwangbo et al., 2004; Carter and Brunet, 2007). In the present study, the injection of *dsLsFoxO* significantly inhibited the gene’s mRNA in the three different nymphal periods and the protein level in the 4th instar nymph (Figure 3B). Compared with these results, the developmental duration of 3rd to 5th instar nymphs may be a better indicator of the functional relationship between *LsFoxO* and nymphal diapause. Diapause is a form of dormancy used by insects to survive under adverse environmental conditions and is usually related to some cold-tolerance substances, such as glycerol, trehalose and glucose, which can improve the insects’ cold tolerance and help overcome severe winter environments (Denlinger, 2002; Zhai et al., 2016). TRE is a key enzyme in trehalose hydrolysis and changes in the activity of this enzyme directly affect energy metabolism (Kamei et al., 2011). PK is the key enzymes in the glycolytic pathway, and PK mediates the conversion of phosphoenolpyruvic acid and ADP into pyruvic acid and ATP (Rider et al., 2011). Here, TRE and PK levels significantly increased after an injection of *dsLsFoxO*, and the glycerol and trehalose levels significantly decreased (Figures 4A,B). Thus, the diapausal nymphs had increased their cold tolerance through the accumulation of cold-tolerance substances.

Targeting a gene using RNAi may reveal its function, and this method has been most recently used for agricultural pest control (Baum et al., 2007; Mao et al., 2007). However, there have been limited studies on the global changes in the mRNA profile that occur after RNAi targeting of a specific gene (Wang et al., 2008). We determined previously that4th instar nymphs represent the main diapausal. Therefore, we selected 1D4N diapausal nymphs to inject with dsRNA and for transcriptomic analyses. We showed that in 384 annotated DEGs between *LsFoxO*-RNAi and control samples, 208 and 176 genes were up- and down-regulated, respectively (Figure 5B). Among the down-regulated genes, we found the RNAi target gene *LsFoxO* (c11725.graph_c0), indicating that the RNAi was effective. To review the global changes in the signaling pathway after RNAi, we used the KEGG analytical method. Most of the DEGs correlated with metabolic and environmental information processes, including carbohydrate metabolism, amino acid metabolism, fatty acid metabolism and the PI3K-Akt signaling pathway (Figure 5C). The latter is a signal transduction pathway that promotes survival and growth in response to extracellular signals and is a key component of the insulin signaling pathway. Insulin activates PI3K-Akt, and high p-Akt levels repress FoxO activity and activate other genes that inhibit diapause.

**FIGURE 6:**
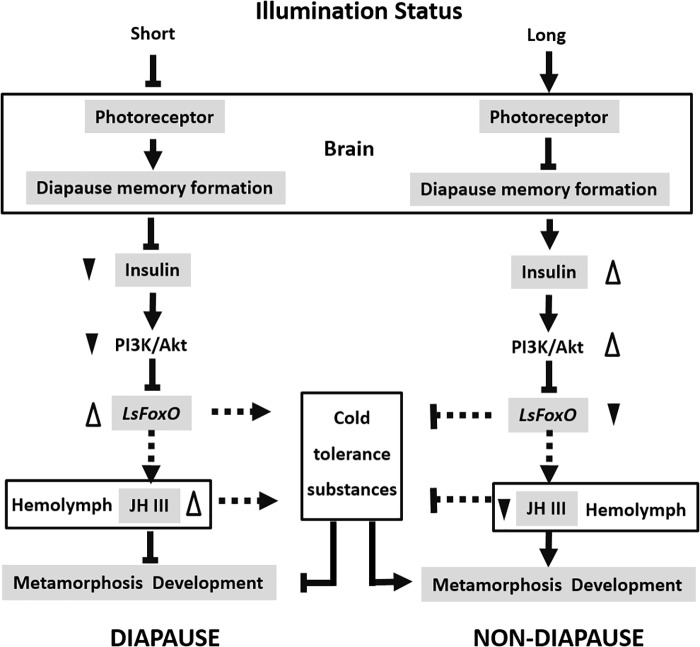
Proposed model of the different photoperiodic signals interacts with *LsFoxO* to regulate the nymphal diapause. Short illumination status decrease photoreceptor activity, and the photoperiodic information will subsequently be stored by a special memory formation process in the brain. The insulin signaling pathway is repressed, which stimulate the *LsFoxO* activity. *LsFoxO* up-regulate some cold tolerance substances accumulation and JH III titers in the hemolymph. Long illumination status results in opposite effects. The model provides an explanation for different photoperiodic signals interacts with *LsFoxO* to regulate the nymphal diapause.

In summary, FoxO is a key downstream regulator that acts as a key developmental switch in insect diapause, which is regulated by the insulin signaling pathway. *LsFoxO* can regulate some cold-tolerance substances and JH III titers in the hemolymph to control the nymphal diapause status. This supports the conclusion that physiological levels of FoxO are beneficial for diapause. We propose a model to explain how different photoperiod signals interact with *LsFoxO* to regulate nymphal diapause in *L. striatellus* (Figure 6). There are still many issues to be studied in the future, such as how *LsFoxO* regulates JH expression and the accumulation of some cold-tolerance substances? The present results offer new insights into nymphal diapause and contribute to a comprehensive understanding of insect diapause.

## Author Contributions

Z-JY, X-LD, YY, and Y-FZ conceived and designed the experiments. Z-JY, KK, G-AW, HC, and XY-D preformed the experiments. Z-JY, LZ, and Y-FZ analyzed the data and wrote the manuscript. All authors read and approved the final manuscript.

## Conflict of Interest Statement

The authors declare that the research was conducted in the absence of any commercial or financial relationships that could be construed as a potential conflict of interest.
