# Supplementary material for: FoxO Transcription Factor Regulate Hormone Mediated Signaling on Nymphal Diapause
Source: Front Physiol. 2018 Nov 20;9:1654. doi: 10.3389/fphys.2018.01654 (PMC6255938; doi:10.3389/fphys.2018.01654)
Supplement: Supplementary file 1 [file Data_Sheet_1.ZIP › Supplementary Data Sheet/Supplementary Figure.docx]

**
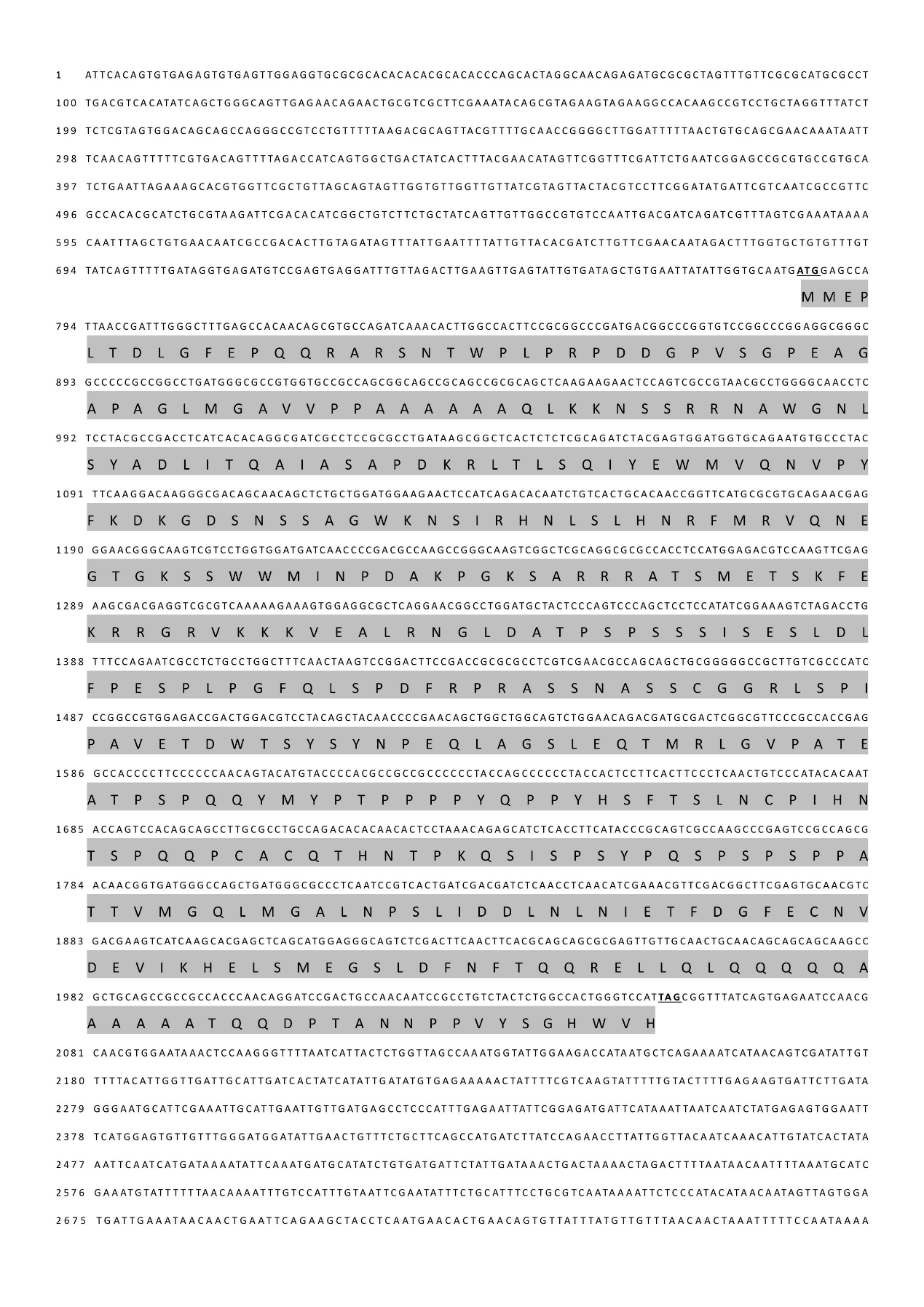
**

**Supplementary Figure 1. Nucleotide and amino acid sequences of *LsFoxO*.** The encoded protein is shaded in gray, underline“-”predicted signal peptide, “*”terminal codon.
