# Supplementary material for: FoxO Transcription Factor Regulate Hormone Mediated Signaling on Nymphal Diapause
Source: Front Physiol. 2018 Nov 20;9:1654. doi: 10.3389/fphys.2018.01654 (PMC6255938; doi:10.3389/fphys.2018.01654)
Supplement: Supplementary file 1 [file Data_Sheet_1.ZIP › Supplementary Data Sheet/Supplementary Tables.docx]

**Supplementary Table 1 Transcriptome sequencing data assembly statistics tables**

| Length Range | Contig | Transcript | Unigene |
| --- | --- | --- | --- |
| 200-300 | 4,715,473(99.72%) | 21,125(32.56%) | 19,578(45.32%) |
| 300-500 | 18,353(0.14%) | 21,141(21.74%) | 15,181(25.33%) |
| 500-1000 | 16,142(0.11%) | 18,914(21.75%) | 14,532(15.26%) |
| 1000-2000 | 13,525(0.06%) | 16,928(16.85%) | 7,548(12.38%) |
| 2000+ | 1,851(0.04%) | 4,251(7.15%) | 3,447(6.22%) |
| Total Number | 4,723,451 | 45,258 | 42,273 |
| Total Length | 212,234,446 | 132,280,943 | 41,547,251 |
| N_50_ Length | 56 | 1,927 | 1,357 |
| Mean Length | 51.39 | 1724.38 | 754.36 |

**Supplementary Table 2 Unigenes annotation database**

| Annotated databases | Unigene | ≥300nt | ≥1000nt |
| --- | --- | --- | --- |
| COG | 10,671 | 9,518 | 5,215 |
| GO | 10,083 | 8,059 | 4,755 |
| KEGG | 9,794 | 8,421 | 4,834 |
| KOG | 18,483 | 17,475 | 9,785 |
| Pfam | 15,526 | 13,214 | 8,237 |
| Swissprot | 9,384 | 7,383 | 5,571 |
| nr | 27,325 | 25,495 | 18,258 |
| All | 31,254 | 28,548 | 14,572 |
